# MTAP Loss and Pleural Mesothelioma (PM): Current Treatment Strategies and Emerging Therapeutic Approaches

**DOI:** 10.3390/cancers18203360

**Published:** 2026-10-17

**Authors:** Susana Cedres, Laura Masfarre, Ilaria Priano, Augusto Valdivia, Pedro Rocha, Patricia Iranzo, Nuria Pardo, Alex Martinez-Marti, Aina Arbusà, Lucia Cane, Mireia Soleda, Barbara Sinigaglia, Sara Polo-Alonso, Gerard Romero-Sola, Rocio Caro-Consuegra, Ana Ortega, Enriqueta Felip

**Affiliations:** 1 Medical Oncology Department, Vall d’Hebron Institute of Oncology (VHIO), Vall d’Hebron Hospital Universitari, 08035 Barcelona, Spain; lauramasfarre@vhio.net (L.M.); amartinezmarti@vhio.net (A.M.-M.); efelip@vhio.net (E.F.); 2 Thoracic Cancers Translational Genomics Unit, Vall d’Hebron Institute of Oncology (VHIO), 08035 Barcelona, Spainbarbarasinigaglia@vhio.net (B.S.); gerardromero@vhio.net (G.R.-S.);

**Keywords:** malignant pleural mesothelioma, biomarkers, MTAP loss, immunotherapy

## Abstract

PM is a locally aggressive tumor associated with asbestos exposure. Despite legislative efforts to regulate asbestos use, its incidence continues to rise in some parts of the world. Chemotherapy and immunotherapy have improved survival in PM patients, but overall survival remains poor. MTAP loss is frequent in PM and may influence tumor behavior, resulting in a poorer prognosis. MTAP-deficient tumors exhibit unique metabolic vulnerabilities that have stimulated the development of targeted therapeutic strategies in recent years with promising results. The aim of this review is to analyze the prognostic and predictive value of MTAP in PM patients, as well as the outcomes of drugs with potential activity against this target.

## Simple Summary

PM is an aggressive malignancy associated with a median survival of approximately two years. Despite substantial efforts to identify actionable molecular alterations, effective targeted therapies remain limited. MTAP loss is a frequent molecular alteration in PM that results in a synthetic lethal vulnerability, providing a potential therapeutic opportunity for the development of MTAP-directed treatment strategies. Current evidence suggests that MTAP loss may represent a marker of poor prognosis in mesothelioma. Recent results from phase 1 studies reveal promising activity in patients with PM. The aim of this review is to discuss the clinical value of MTAP loss in PM and future targeted therapies.

## 1. Introduction

Pleural mesothelioma (PM) is a rare and highly aggressive malignancy primarily caused by asbestos exposure. Although mesothelioma arises predominantly from the pleural lining, it can also develop in the peritoneum, pericardium, or tunica vaginalis.

According to the SEER database, the incidence of PM in 2023 was approximately 0.7 cases per 100,000 person-years [1]. Over the last few decades, incidence rates have remained stable among women while progressively declining in men.

Despite its relatively low incidence, PM remains a major clinical challenge because of its poor prognosis and limited therapeutic options. Platinum-based chemotherapy combined with pemetrexed has been the standard first-line treatment for nearly two decades [2]. More recently, immunotherapy has changed the therapeutic landscape. The combination of nivolumab and ipilimumab demonstrated a significant overall survival benefit in the phase III CheckMate 743 trial, while the addition of pembrolizumab to platinum-pemetrexed chemotherapy also showed improved efficacy in the phase III KEYNOTE-483 trial [3,4].

The pathological diagnosis of PM still relies on tissue biopsy demonstrating both mesothelial differentiation and evidence of invasion [5]. Histologically, PM is classified into three major subtypes (epithelioid, sarcomatoid, and biphasic), each with different prognostic and predictive significance. The current WHO classification recognizes these three histological variants and incorporates mesothelioma in situ (MIS) as a distinct diagnostic entity [5]. Recent genomic studies support the need for a more refined and clinically meaningful classification beyond the traditional histological subtypes. In addition, the combined assessment of BAP1 and CDKN2A/MTAP has emerged as a valuable tool for establishing the diagnosis of epithelioid mesothelioma [6].

The genomic landscape of PM is characterized predominantly by the loss of tumor suppressor genes. The most frequently altered genes in PM are BAP1, CDKN2A, CDKN2B, NF2, MTAP, TP53 and SETD2 [7]. Since these genes function mainly as tumor suppressors, they are inherently difficult to target therapeutically.

Among the molecular alterations identified in PM, the loss of methylthioadenosine phosphorylase (MTAP) has emerged as a biomarker of interest in mesothelioma research in diagnosis and as a promising therapeutic marker. MTAP is a key enzyme in the methionine salvage pathway, catalyzing the conversion of 5′-methylthioadenosine into adenine and 5-methylthioribose-1-phosphate, thereby contributing to polyamine metabolism, nucleotide homeostasis, and cellular methylation processes [8]. Homozygous deletion or reduced expression of MTAP, often resulting from its close genomic proximity to CDKN2A on chromosome 9p21, occurs in approximately 40–70% of mesotheliomas, depending on the histological subtype and detection method [9,10]. Because MTAP loss closely correlates with CDKN2A deletion, immunohistochemical (IHC) assessment of MTAP has become a valuable surrogate marker that complements fluorescence in situ hybridization (FISH) in distinguishing mesothelioma from reactive mesothelial proliferations [9,11,12].

MTAP loss may influence tumor behavior, although its prognostic significance remains controversial across different patient cohorts [13,14,15,16,17]. MTAP-deficient tumors exhibit unique metabolic vulnerabilities that have stimulated the development of targeted therapeutic strategies. These include inhibition of protein arginine methyltransferase 5 (PRMT5), methionine adenosyltransferase 2A (MAT2A), and other components of metabolic pathways selectively required by MTAP-deficient cells, several of which are currently under preclinical or clinical investigation [18,19,20,21,22,23].

This review summarizes the current understanding of MTAP biology, its prevalence and clinical significance in mesothelioma, its diagnostic, prognostic, and predictive value, and the emerging therapeutic approaches aimed at exploiting MTAP deficiency.

## 2. MTAP Gene

The MTAP gene is a tumor suppressor located on chromosome 9p21.3 [24], a genomic region that undergoes one of the most frequent homozygous deletions across human cancers. Owing to its close proximity to CDKN2A, MTAP is commonly co-deleted with this gene, and loss of chromosome 9p21 is observed in the majority of pleural mesotheliomas [24,25,26].

MTAP is ubiquitously expressed in normal tissues and encodes methylthioadenosine phosphorylase, a key enzyme in the methionine and purine salvage pathways. MTAP catalyzes the phosphorolysis of 5′-methylthioadenosine (MTA) into adenine and 5-methylthioribose-1-phosphate [27]. The generated adenine is recycled for AMP synthesis, whereas 5-methylthioribose-1-phosphate is further metabolized through the methionine salvage pathway to regenerate methionine [28]. Through these functions, MTAP maintains cellular pools of adenine and methionine, thereby supporting cell proliferation, differentiation, inflammatory responses, gene expression, and apoptosis [29].

**Figure 1:**
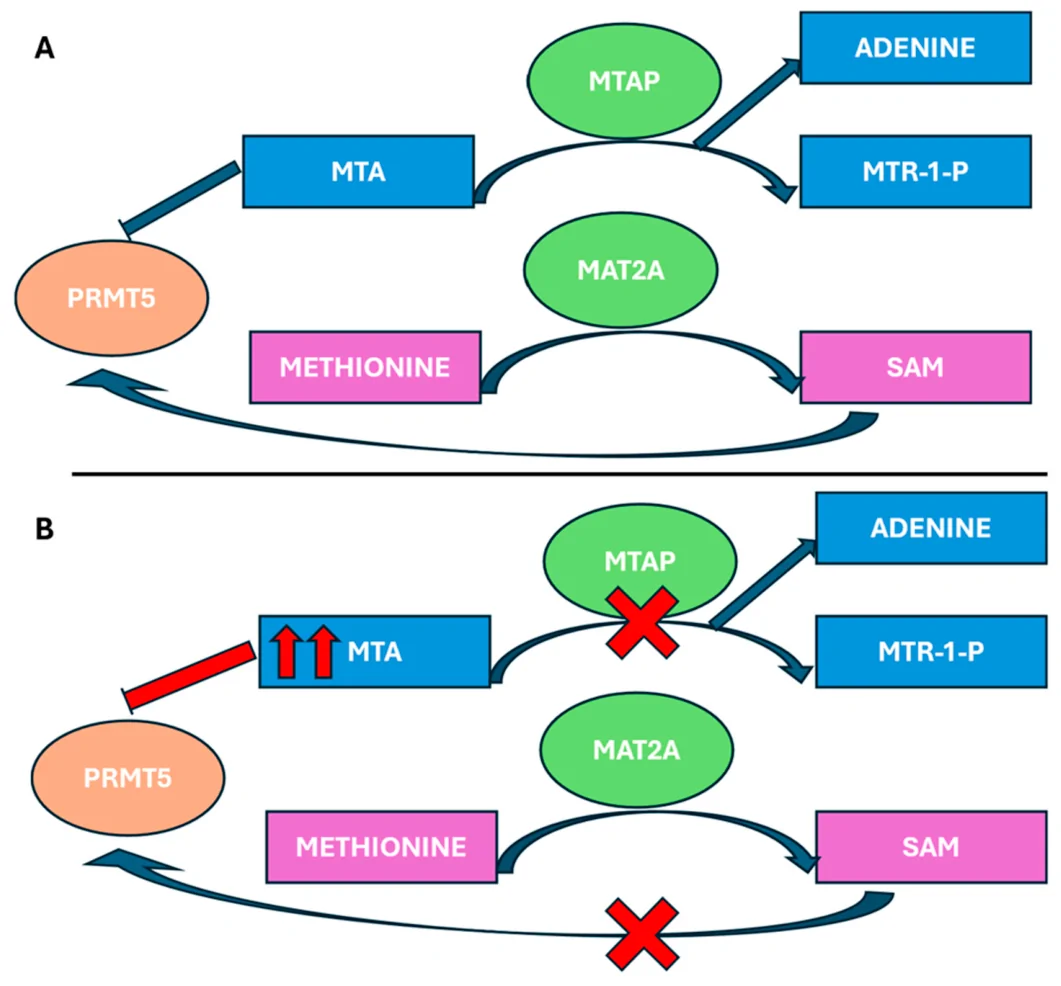
Methionine salvage pathway in MTAP wild-type (**A**) and in MTAP-null cells (**B**).

Loss of MTAP disrupts these metabolic pathways, resulting in intracellular accumulation of MTA. Because MTA competes with S-adenosylmethionine (SAM) for binding to PRMT5, elevated MTA levels partially inhibit PRMT5 activity, but complete inhibition of PRMT5 is incompatible with the survival of most cell types [18,19]. Consequently, MTAP-deficient tumor cells become highly dependent on the residual PRMT5 activity required to maintain essential cellular functions. MTAP loss therefore renders cancer cells particularly sensitive to pharmacological inhibition of the PRMT5 pathway (Figure 1) [18]. This selective dependency represents a classic example of synthetic lethality and has established PRMT5 as an attractive therapeutic target in tumors harboring homozygous MTAP deletion [18,19].

Loss of MTAP alters cellular metabolism, epigenetic regulation, and immune signaling, thereby contributing to tumorigenesis and immune evasion [18,30,31]. In MTAP-proficient cells, MTAP maintains the balance between metabolic and epigenetic pathways by increasing adenine availability while preventing MTA accumulation, thereby favoring formation of the active PRMT5–SAM complex over the inactive PRMT5–MTA complex [18,19,31]. Conversely, in MTAP-deficient tumors, MTA accumulation within the tumor microenvironment contributes to immune suppression by impairing T-cell and natural killer cell function. Consistent with these observations, MTAP-deleted tumors have been associated with an immunologically “cold” tumor microenvironment and reduced responsiveness to immune checkpoint blockade.

## 3. Mesothelioma in Situ and Well-Differentiated Papillary Mesothelial Tumor

The 2021 World Health Organization (WHO) Classification introduced major changes in the classification of preinvasive mesothelial lesions. These included the recognition of MIS as a distinct diagnostic entity and the revision of the nomenclature and diagnostic criteria for well-differentiated papillary mesothelial tumor (WDPMT) [5].

MIS is currently classified as a preinvasive mesothelial neoplasm characterized by a single layer of cytologically bland mesothelial cells lining the pleural surface, without evidence of stromal invasion. The diagnosis requires demonstration of loss of BAP1 and/or MTAP expression by IHC and/or homozygous CDKN2A (p16) deletion by FISH. By definition, there should be no radiological or macroscopic evidence of an established tumor, and the presence of invasive mesothelioma must be excluded before a diagnosis of MIS can be made [5,32,33].

In contrast, although immunohistochemical and molecular analyses are recommended in the evaluation of WDPMT, they are not considered mandatory for its diagnosis. However, increasing evidence suggests that alterations in BAP1, MTAP, and CDKN2A may identify lesions with a higher risk of progression and may warrant reclassification.

In a small series of 14 peritoneal WDPMTs evaluated for MTAP/CDKN2A, three tumors showed loss of MTAP/CDKN2A expression, while loss of BAP1 expression was identified in 9 of 21 tumors analyzed. Among patients who subsequently progressed to invasive mesothelioma, all tumors demonstrated BAP1 loss, whereas the two cases evaluated for MTAP retained MTAP expression. Importantly, none of the seven tumors with retained BAP1 and MTAP/CDKN2A expression progressed to invasive mesothelioma [34]. Based on these findings, the authors proposed that retained BAP1 expression should be a prerequisite for the diagnosis of WDPMT and that lesions showing BAP1 loss should not be classified as WDPMT. Owing to the limited number of cases, the clinical significance of isolated MTAP loss could not be determined. A scoping review also concluded that WDPMT exhibiting BAP1 and/or MTAP loss should be classified as papillary MIS [35].

## 4. Diagnostic and Histopathology

### 4.1. Diagnostic Techniques of MTAP

The current classification of pleural tumours includes the incorporation of nuclear grading, architectural patterns, cytological and stromal features for epithelioid diffuse pleural mesothelioma, as well as the recognition of MIS as a distinct diagnostic entity. In this context, demonstration of BAP1 or MTAP loss by IHC, or homozygous CDKN2A deletion by FISH, has become an important ancillary tool for the diagnosis of epithelioid mesothelioma (Table 1) [5].

The diagnostic utility of MTAP immunohistochemistry has been particularly well established in the distinction between reactive mesothelial proliferations and mesothelioma [12,27,36,37]. Loss of MTAP expression has consistently demonstrated excellent specificity for malignant mesothelioma, generally approaching 100%, whereas sensitivity ranges from approximately 40% to 65% depending on the study population and methodology [38,39,40,41].

Interpretation of MTAP immunohistochemistry requires careful assessment of both staining quality and internal positive controls. Although complete loss of both nuclear and cytoplasmic staining is typically observed, cytoplasmic loss is generally the most evident and diagnostically useful finding [39]. Nuclear and cytoplasmic loss have been shown to be fully concordant in pleural effusion cytology [42].

Scoring MTAP may be complicated by heterogeneous staining resulting from preanalytical variables, including fixation and tissue processing, as well as differences in antigen retrieval. In addition, antibody selection influences diagnostic performance. Comparative studies have demonstrated that the monoclonal antibody clone 1813 provides stronger staining intensity and fewer equivocal cases compared with clone EPR6893 [43].

MTAP immunohistochemistry has been incorporated into current diagnostic guidelines for PM. Nevertheless, its implementation in routine clinical practice remains limited. For example, an Italian nationwide survey reported that MTAP immunohistochemistry was routinely performed in only 30% of participating institutions [44].

While MTAP IHC is increasingly recommended for patient stratification in clinical trials investigating PRMT5 inhibitors, its translation into routine practice faces a significant unmet need for technical standardization. Currently, protocols vary across institutions regarding antibody clone selection (e.g., EPR6893 vs 1813), definition of optimal cut-off thresholds, and the interpretation of discordant nuclear versus cytoplasmic staining patterns. Recent updates from international expert panels, such as the 2024 IMIG consensus statement, have validated MTAP loss as a surrogate for CDKN2A homozygous deletion; however, establishing a universal scoring consensus remains mandatory to prevent false negatives driven by tissue fixation artifacts or tumor-infiltrating immune cells.

Although next-generation sequencing (NGS) is increasingly used to identify MTAP deletions, there is currently no consensus regarding optimal thresholds for defining copy-number loss. Nevertheless, accurate identification of MTAP deletion by large-panel NGS has become increasingly relevant for patient selection in ongoing clinical trials targeting MTAP-deficient tumors [45].

Importantly, the diagnostic and therapeutic applications of MTAP testing should be distinguished. MTAP immunohistochemistry is primarily a diagnostic ancillary test and a practical surrogate for CDKN2A homozygous deletion, particularly in PM. However, loss of MTAP protein expression does not necessarily establish the presence of a genomic MTAP deletion. Therefore, when MTAP status is being assessed for eligibility for a targeted therapy trial, molecular confirmation of the genomic alteration should be considered according to the specific trial inclusion criteria. In this setting, NGS, MTAP-specific FISH, or another validated molecular assay may be required to confirm MTAP deletion, whereas MTAP immunohistochemistry alone may be sufficient for diagnostic purposes.

### 4.2. Co-Deletion of CDKN2A and MTAP

Immunohistochemical detection of cytoplasmic MTAP loss has been extensively investigated as a surrogate marker for homozygous CDKN2A deletion detected by FISH [39,40,41]. Because MTAP is located adjacent to CDKN2A, homozygous MTAP deletion is almost invariably accompanied by CDKN2A deletion and approximately 75–90% of PM harboring homozygous CDKN2A deletion also exhibit concomitant deletion of MTA [9,11,12].

Multiple studies have demonstrated a high concordance between MTAP protein loss by IHC and CDKN2A homozygous deletion detected by FISH [38,39,46,47,48]. Brcic and colleagues demonstrated that cytoplasmic MTAP loss identified CDKN2A homozygous deletion with high accuracy when a cut-off of 30% positive tumor cells was applied [49]. Diagnostic performance can be further improved by combining MTAP immunohistochemistry with other markers. In particular, the combined assessment of loss of nuclear p16 expression (cut-off ≤ 1%) and loss of cytoplasmic MTAP expression (cut-off ≥ 30%) achieved a specificity of 96% and a sensitivity of 86% for predicting CDKN2A homozygous deletion [50]. Although MTAP alone has only moderate sensitivity, its diagnostic performance improves substantially when combined with BAP1 immunohistochemistry and/or CDKN2A FISH analysis [25,51,52,53]. In a small series of 10 PM cases, CDKN2A homozygous deletion was identified in 30% of tumors, and all deleted cases showed concomitant loss of cytoplasmic MTAP expression, resulting in 100% concordance [54]. Similarly, a large multi-institutional study including 99 PM cases demonstrated that MTAP loss detected by immunohistochemistry was 78% sensitive and 96% specific for identifying CDKN2A homozygous deletion [25]. In a subsequent study, the same investigators confirmed that MTAP immunohistochemistry is a highly specific, although only moderately sensitive, surrogate marker for CDKN2A deletion.

Complete loss of MTAP expression is almost exclusively associated with homozygous deletion, whereas CDKN2A microdeletions or focal deletions may occur with preserved MTAP expression [55]. Although contradictory data have suggested MTAP deficiency in a proportion of tumors with heterozygous 9p21 deletion, complete MTAP loss is generally considered to reflect homozygous deletion [48]. However, the converse is not always true; therefore, CDKN2A loss should not be considered a reliable surrogate marker for MTAP deletion [16].

Despite these findings, MTAP cannot be regarded as a universal surrogate for CDKN2A deletion in all mesothelioma subtypes. In particular, studies in peritoneal mesothelioma have reported lower concordance between MTAP expression and CDKN2A status, indicating that MTAP immunohistochemistry should be interpreted cautiously outside the pleural setting [25,49,56].

Several methodologies have been developed to detect chromosome 9p21 deletion, including FISH, IHC, NGS and droplet digital PCR (ddPCR). Among these approaches, FISH remains the reference method for detecting CDKN2A homozygous deletion, whereas MTAP immunohistochemistry offers a rapid, cost-effective, and widely available alternative based on the loss of a surrogate protein marker [12].

### 4.3. Histological and Cytological Applications

The diagnostic utility of MTAP extends beyond tissue biopsies to cytological specimens. Several studies have reported excellent concordance between MTAP expression in pleural effusion cell blocks and matched histological specimens [36,37,41,57,58,59,60,61,62].

Reported rates of MTAP loss in pleural effusions range from 33% to 76%, depending on the study cohort and staining methodology [57,60,63,64,65]. Complete loss of MTAP expression shows excellent specificity for mesothelioma in cytological samples, although sensitivity remains lower than in tissue biopsies, ranging from approximately 38% to 44% in cytology and 42% to 55% in histological specimens [39,53,59].

In one study including paired pleural biopsies and pleural effusion specimens, overall concordance for MTAP expression reached 72%, while several additional studies reported complete concordance between cytological and histological samples [37,57,58,59,60,63,64]. Furthermore, complete loss of MTAP staining appears to be a reliable indicator of malignancy, whereas the significance of partial loss remains uncertain [60].

MTAP immunohistochemistry has also shown diagnostic value in sarcomatoid mesothelioma, where it effectively distinguishes sarcomatoid mesothelioma from fibrous pleuritis. Sensitivity reached 80% with MTAP alone and increased to 90% when combined with BAP1 immunohistochemistry [64].

## 5. Prevalence of MTAP Loss in Mesothelioma

MTAP deletion is one of the most frequent molecular alterations in mesothelioma, although its reported prevalence varies depending on the anatomical site, histological subtype, and detection method used. Overall, genomic studies have reported MTAP alterations in approximately 27–40% of mesotheliomas, whereas immunohistochemical studies generally identify loss of MTAP protein expression in 30–60% of cases [8,9].

A large retrospective clinicogenomic analysis including 10,936 patients tested with the FoundationOne CDx assay identified homozygous MTAP deletion in 9.4% of all cancers, with mesothelioma representing one of the tumor types most frequently affected, showing MTAP deletion in 40.5% of cases [26]. Similarly, an analysis of 1113 pleural and peritoneal mesotheliomas profiled by FoundationOne reported MTAP alterations in 27% of tumors [10].

Different studies have reported variable frequencies of MTAP loss. In a large pan-cancer study including 17,078 tumors from 149 different tumor types, complete loss of MTAP expression was identified in 32% of mesotheliomas. Importantly, all mesothelioma cases with complete MTAP loss by immunohistochemistry showed complete concordance with homozygous deletion confirmed by FISH. Across all tumor types, complete MTAP loss was also associated with higher PD-L1 expression [66].

The prevalence of MTAP loss differs according to histological subtype. Loss of chromosome 9p21 and MTAP expression is more frequent in sarcomatoid than in epithelioid mesothelioma, reflecting the greater prevalence of CDKN2A homozygous deletion in sarcomatoid tumors [5,9,25,36]. Cytoplasmic MTAP loss has been reported in approximately 40% of epithelioid pleural mesotheliomas compared with up to 75% of sarcomatoid tumors, while CDKN2A homozygous deletion occurs in approximately 60% and 93% of these subtypes, respectively [9,11,36,67].

In biphasic mesothelioma, MTAP expression may be heterogeneous, with retained staining in the epithelioid component and loss in the sarcomatoid component. Consequently, current recommendations consider loss of BAP1 or MTAP in the spindle-cell component as supportive evidence of malignancy. Conversely, when loss is confined to the epithelioid component, the diagnosis of biphasic mesothelioma should only be established if the spindle-cell component also demonstrates unequivocal morphological features of malignancy [10].

Differences in MTAP prevalence have also been observed according to anatomical site. In a study including 60 pleural and 53 peritoneal mesotheliomas, complete or partial loss of MTAP expression was significantly more frequent in pleural than in peritoneal mesothelioma (35/60 vs. 11/53) [68]. Similarly, in a small series of peritoneal mesotheliomas, loss of MTAP expression was observed in 28% of epithelioid tumors and 33% of biphasic tumors [69].

Overall, the reported prevalence of MTAP loss ranges widely across studies, reflecting differences in patient cohorts, histological subtype, anatomical location, and the methodology used to assess MTAP status. Nevertheless, the available evidence consistently indicates that MTAP loss is a common event in mesothelioma, particularly in pleural and sarcomatoid tumors, supporting its biological and clinical relevance [68,70].

## 6. Prognostic Significance of MTAP Loss

Homozygous MTAP deletions are frequent across human cancers and have generally been associated with aggressive tumor biology and poor clinical outcomes [13,14,15].

Several studies have suggested that MTAP loss is associated with shorter survival in mesothelioma [16,71]. A retrospective analysis evaluating the prognostics of MTAP across multiple tumor types demonstrated that MTAP-deficient tumors had reduced overall survival (OS), with a particularly pronounced effect in mesothelioma patients. In this cohort, median OS was 12.8 months in MTAP-loss mesotheliomas compared with 23.0 months in MTAP-proficient tumors. Interestingly, MTAP-deficient tumors showed lower rates of TP53 mutations, yet the survival disadvantage persisted, suggesting that the adverse prognostic effect of MTAP loss may be independent of TP53 status [26].

Similarly, Busacca et al. reported that loss of MTAP expression was associated with disease progression and represented a negative prognostic factor in pleural mesothelioma, with MTAP-deficient patients showing significantly shorter OS compared with patients retaining MTAP expression [72].

In a cohort including both pleural and peritoneal mesotheliomas, MTAP loss was associated with a trend toward shorter OS. However, the association reached statistical significance when the analysis was restricted to pleural mesothelioma patients, suggesting that the prognostic impact of MTAP loss may differ according to anatomical site [68].

Genomic studies have provided additional evidence supporting a potential prognostic role for MTAP deletion. In a series of 79 mesothelioma patients treated with radical surgery, MTAP deletion was identified in 42% of cases, and patients with heterozygous or homozygous MTAP deletion had shorter median OS compared with MTAP wild-type tumors (12.5 vs. 17.6 months). These findings were validated in an independent cohort of 100 samples, where MTAP deletion was detected in 63% of patients and was associated with significantly shorter OS (8.7 vs. 22.7 months) [73].

However, not all studies have confirmed an independent prognostic role for MTAP. A large-scale genomic characterization of Japanese pleural mesothelioma patients demonstrated that TP53 mutations were significantly associated with shorter survival, whereas alterations in BAP1, NF2, CDKN2B, and MTAP were not independently associated with OS. Given the frequent co-occurrence between MTAP and CDKN2A loss, the authors also evaluated the overlap between these alterations and observed a tendency toward earlier survival decline in patients with CDKN2A loss alone rather than combined MTAP/CDKN2A loss [74]. However, a combined analysis was not performed in this study to evaluate the potential prognostic impact of MTAP loss in the context of TP53 mutations.

The biological mechanisms underlying the potential prognostic impact of MTAP loss remain incompletely understood. MTAP-deficient tumors accumulate MTA, resulting in partial inhibition of PRMT5 activity and altered regulation of RNA splicing, DNA repair, and epigenetic processes. Conversely, increased PRMT5 expression has been associated with tumor progression and poor prognosis across multiple malignancies, including breast cancer, glioblastoma, prostate cancer, pancreatic cancer, bladder cancer, leukemia, lymphoma, ovarian cancer, lung cancer, and gastric cancer [74].

Overall, current evidence suggests that MTAP loss may represent a marker of aggressive tumor biology and poor prognosis in mesothelioma, particularly in pleural disease. Nevertheless, the prognostic value of MTAP requires further validation in larger prospective cohorts, ideally incorporating comprehensive molecular profiling and adjustment for established prognostic factors.

## 7. Predictive Value of MTAP Loss

Beyond its diagnostic and therapeutic implications, MTAP loss has emerged as a potential biomarker with predictive relevance. However, available evidence is mainly derived from retrospective analyses and studies across different tumor types.

### 7.1. MTAP Loss and Chemotherapy Response

The predictive role of MTAP status in response to conventional chemotherapy remains controversial. A real-world genomic analysis including multiple tumor types reported a significantly shorter median time to next treatment among patients with MTAP deletions receiving chemotherapy compared with MTAP-proficient tumors. However, among patients treated with chemo-immunotherapy combinations, no significant difference in time to next therapy was observed according to MTAP status. In the subgroup receiving pemetrexed-based chemotherapy, no significant difference was observed between MTAP-deleted and MTAP-proficient tumors (median time to next treatment: 6.9 vs. 8.5 months) [26].

Conversely, some studies suggest that MTAP loss may increase sensitivity to antifolate-based therapies. In a small phase II trial in urothelial carcinoma, MTAP-deficient tumors showed higher response rates to pemetrexed compared with MTAP-proficient tumors. However, in a retrospective NSCLC cohort, patients with MTAP-proficient tumors demonstrated significantly better responses to carboplatin plus pemetrexed combination therapy. These conflicting findings indicate that the predictive value of MTAP status for pemetrexed sensitivity remains uncertain and requires further investigation.

### 7.2. MTAP Loss and Response to Immune Checkpoint Inhibitors

Emerging evidence indicates that MTAP deficiency may contribute to an immunosuppressive tumor microenvironment and may be associated with diminished responsiveness to immune checkpoint inhibitors [26].

The proposed mechanism underlying this association involves the accumulation of methylthioadenosine (MTA) within MTAP-deficient tumors. Increased extracellular and intracellular MTA levels contribute to immune suppression through inhibition of PRMT5 activity and activation of adenosine-related signaling pathways, impairing the function of CD8^+^ and CD4^+^ T lymphocytes. Consistent with this mechanism, MTAP-deficient tumors show reduced lymphocytic infiltration and features of an immunologically “cold” tumor microenvironment in preclinical models [75,76,77].

The association between MTAP loss and resistance to immunotherapy has been described in other malignancies. Homozygous deletion of chromosome 9p21 has been associated with poor outcomes in patients treated with anti-PD-L1 monotherapy, independently of PD-L1 expression and tumor mutational burden (TMB). This effect appears to be more pronounced in patients receiving immune checkpoint inhibitor monotherapy compared with combination immunotherapy approaches [75]. In breast cancer, PD-L1 expression was significantly lower in MTAP-deficient tumors compared with MTAP-intact tumors, whereas in non-small cell lung cancer (NSCLC), TMB was significantly higher in MTAP-proficient tumors than in MTAP-loss tumors. These findings suggest that MTAP deficiency may define a biologically distinct subgroup characterized by reduced immune activation [78].

In PM, the evidence supporting MTAP as a factor of resistance to immunotherapy stems from retrospective analysis [79,80]. A first study with 22 mesothelioma patients treated with nivolumab found no statistically significant difference in PFS between patients with MTAP loss and those with retained MTAP expression (4.6 vs. 5.7 months, respectively). Similarly, differences in overall survival did not reach statistical significance. Furthermore, another study evaluating the predictive value of several biomarkers in 113 patients with pleural or peritoneal mesothelioma found that although specimens with MTAP loss exhibited an immune-desert immunophenotype, there were no significant differences in treatment efficacy with nivolumab plus ipilimumab (PFS 2.6 versus 5.6 months and OS 30.5 m vs not reached for MTAP loss vs MTAP retained, both no significant).

## 8. Therapeutic Strategies Targeting MTAP in Mesothelioma

The high prevalence of MTAP loss in pleural mesothelioma has prompted the development of therapeutic strategies that exploit the unique metabolic dependencies of MTAP-deficient tumors (Table 2).

The first therapeutic strategy specifically explored in MTAP-deficient mesothelioma targeted the altered purine and methionine metabolism associated with MTAP deficiency. L-alanosine, an inhibitor of adenylosuccinate synthetase involved in de novo adenine nucleotide synthesis, in preclinical studies suggested increased sensitivity of MTAP-negative tumors. However, a phase II clinical trial failed to demonstrate objective responses, indicating limited clinical activity [66,79]. Although the negative results of trial, L-alanosine provided proof-of-concept for synthetic lethal strategies targeting MTAP-deficient cancers.

More recently, quinacrine, an antimalarial agent, has shown to impair the survival of MTAP-null cells through mechanisms involving disruption of autophagy, metabolic stress, and inhibition of adaptive survival pathways, thereby enhancing the therapeutic vulnerability conferred by MTAP loss. Quinacrine, in preclinical studies, has shown to suppress PRMT5 transcription, reducing clonogenic growth in MTAP-deficient cells through a PRMT5-dependent mechanism [72].

Unlike the diagnostic use of MTAP immunohistochemistry, in which MTAP loss may be used as a surrogate marker of CDKN2A deletion, eligibility for MTAP-targeted treatment may require demonstration of a genomic MTAP deletion or other molecularly defined MTAP-deficient state. Accordingly, MTAP immunohistochemical loss should not automatically be considered sufficient evidence of eligibility for targeted therapy, and molecular confirmation should be performed when required by the study protocol.

### 8.1. PRMT5 Inhibition

Preclinical studies further support PRMT5 as a therapeutic target in mesothelioma. In MTAP-deficient mesothelioma cell lines, selective PRMT5 inhibition inhibits cell proliferation, induces cell-cycle arrest and apoptosis, and downregulates genes associated with epithelial–mesenchymal transition [20]. Similarly, exogenous MTA administration and stable PRMT5 knockdown selectively impair the growth of MTAP-deleted mesothelioma cells, confirming the biological dependence of these tumors on residual PRMT5 activity [20].

The first generation of PRMT5 inhibitors, including JNJ-64619175, GSK3326595, PF-06939999, PRT543, and PRT811, efficiently inhibited PRMT5 activity in preclinical models. However, early clinical trials in patients with solid tumors and hematological malignancies demonstrated limited efficacy because dose escalation was constrained by hematologic and hepatic toxicities, reflecting the essential role of PRMT5 in normal tissues [21,81,82].

To overcome these limitations, a new generation of MTA-cooperative PRMT5 inhibitors has been developed. These compounds selectively target the inactive PRMT5–MTA complex that accumulates in MTAP-deficient cells, thereby enhancing tumor selectivity and minimizing toxicity in normal tissues. Among these agents, MRTX1719/BMS-986504 (navlimetostat) has shown the most advanced clinical development. MRTX1719 selectively stabilizes the PRMT5–MTA complex and inhibits the residual PRMT5 activity present in MTAP-deficient tumors [12]. Preclinical studies demonstrated potent antitumor activity across multiple MTAP-deleted xenograft models, including mesothelioma, while showing minimal activity in MTAP-proficient tumors [21].

Early clinical results have also been encouraging. In an ongoing phase I/II study (NCT05245500), MRTX1719 produced confirmed objective responses in patients with advanced MTAP-deleted solid tumors and demonstrated a favorable safety profile [21]. The phase I study included two patients with PM, both of whom achieved meaningful reductions in tumor burden. One patient with epithelioid PM harboring an MTAP deletion identified by NGS, who had previously undergone pleurectomy/decortication followed by cisplatin–pemetrexed and nivolumab–ipilimumab, achieved a partial response and remained on treatment for more than eight cycles. The second patient, also with epithelioid PM, had previously received carboplatin–pemetrexed followed by nivolumab combined with a WT1 vaccine and likewise achieved a partial response, with a 30% reduction in target lesions that was maintained for more than eight cycles. In both cases, treatment was well tolerated, with elevated pancreatic enzymes and diarrhea representing the most frequently reported treatment-related adverse events. The trial is currently ongoing and remains open for the enrollment of patients with solid tumors [22,74].

Another MTA-cooperative PRMT5 inhibitor under clinical evaluation is AMG 193 (anvumetostat), a CNS-penetrant methylthioadenosine-cooperative PRMT5 inhibitor, which selectively induces synthetic lethality in MTAP-deleted tumor cells. The phase I trial enrolled 80 patients with advanced CDKN2A-deleted and/or MTAP-deleted solid tumors, including three patients with PM. Of the three patients with PM, two achieved stable disease, while the remaining patient had progressive disease as the best overall response [83]. To date, no further clinical studies have been conducted to evaluate this agent in patients with PM.

TNG908 is a clinical-stage PRMT5 inhibitor that binds cooperatively with MTA and is designed to exploit the synthetic lethal interaction between PRMT5 inhibition and MTAP deletion. In preclinical xenograft models, TNG908 induced sustained tumor regression [84]. The agent is currently under investigation in an ongoing phase I/II clinical trial enrolling patients with advanced solid tumors, including patients with PM, NCT05275478.

### 8.2. MAT2A Inhibition

An alternative synthetic lethal strategy targets MAT2A, the enzyme responsible for synthesizing SAM, the methyl donor required by PRMT5. Pharmacological inhibition of MAT2A decreases intracellular SAM levels, further impairing PRMT5 function and selectively inhibiting the growth of MTAP-deficient tumor cells [12].

Several MAT2A inhibitors have entered clinical development. AG-270, an oral potent, reversible inhibitor of MAT2A, demonstrated promising preclinical activity and has been evaluated in a phase I clinical trial. The study included 4 patients with PM, but no responses were reported [85]. Likewise, IDE397 has advanced into clinical testing after showing potent antitumor activity in MTAP-deficient xenograft models (NCT04794699). Currently, it is being evaluated in a phase 1 trial for patients with MTAP tumors including the inclusion of PM [23].

### 8.3. Hippo Pathway Inhibition

MTAP-deficient tumors may exhibit increased dependence on the Hippo pathway. Preliminary evidence suggests that inhibition of TEAD signaling may represent another therapeutic opportunity in MTAP-deficient mesothelioma, although this strategy remains at an early stage of investigation. More recently, the preliminary results of the TEADES study were presented at the ASCO Annual Meeting, evaluating ODM-212, a small-molecule pan-TEAD inhibitor, in patients with advanced solid tumors [24]. The study enrolled 76 patients, including 29 with PM. Overall, the treatment demonstrated a favorable safety profile and was generally well tolerated. The overall response rate (ORR) across all tumor types was 15%; however, more encouraging activity was observed in the mesothelioma cohort, where an ORR of 27.8% was reported among the first 18 evaluable patients. These preliminary findings suggest that pan-TEAD inhibition may represent a promising therapeutic strategy for patients with PM and warrant further clinical investigation.

### 8.4. Cell-Cycle Inhibition

Loss of CDKN2A, which frequently accompanies MTAP deletion, has also provided the rationale for evaluating CDK4/6 inhibitors in mesothelioma. In the phase II MiST2 trial, abemaciclib achieved a 12-week disease control rate of 54% in patients with p16-deficient pleural mesothelioma [86]. Interestingly, a post hoc exploratory analysis showed loss of MTAP expression in 44% of tumors, and none of the patients whose best response was progressive disease exhibited MTAP loss. Moreover, MTAP-deficient tumors experienced significantly greater reductions in tumor volume following abemaciclib treatment, suggesting that MTAP status may represent a predictive biomarker for CDK4/6 inhibition.

To date, no therapies have been specifically approved for the treatment of MTAP-deficient mesothelioma. Nevertheless, MTAP loss has emerged as one of the most promising therapeutic vulnerabilities in this disease. Loss of MTAP results in intracellular accumulation of MTA, which partially inhibits PRMT5. Consequently, MTAP-deficient tumor cells become highly dependent on the residual PRMT5 activity required to maintain essential cellular functions, creating a synthetic lethal vulnerability that can be therapeutically exploited [9,18,38].

## 9. Discussion

PM is a locally aggressive disease associated with asbestos exposure and the incidence continues to rise in some parts of the world. The prognosis of PM varies, with survival rates generally below two years. Immunotherapy has been established as a new first-line treatment option, improving survival by approximately four months compared to chemotherapy. However, not all patients benefit from immunotherapy, highlighting the unmet need for predictive treatment markers.

Over the past decades, both molecular studies and clinical trials exploring new therapies for PM patients have intensified. However, the results remain largely disappointing. Unlike other thoracic tumors, somatic mutations in PM are rare. MTAP loss represents a relevant molecular alteration in PM, where it is frequently associated with 9p21/CDKN2A co-deletion. MTAP deletion has been reported in approximately 40% of patients with PM, making mesothelioma one of the tumor types with the highest prevalence of MTAP loss. It appears to be more frequent in tumors with sarcomatoid histology, with reported rates of up to 75%. Furthermore, MTAP deletion is more frequently observed in PM than in peritoneal mesothelioma.

MTAP has attracted increasing attention because its loss links a recurrent genomic alteration to distinct biological consequences and potentially actionable therapeutic vulnerabilities (Table 3). However, the clinical significance of MTAP deficiency extends beyond its association with CDKN2A deletion or its emerging role as a therapeutic target. A central challenge is to determine how MTAP status should be interpreted across different clinical settings and how its biological relevance can be translated into clinically validated applications.

From a diagnostic perspective, until recently, FISH was considered the gold standard for detecting CDKN2A homozygous deletion. However, because FISH is technically demanding, time-consuming, and costly, MTAP IHC has emerged as a practical and cost-effective surrogate marker. Owing to the close proximity of MTAP and CDKN2A (approximately 100 kb apart) on chromosome 9p21, homozygous co-deletion occurs in up to 90% of PM, providing the biological basis for using MTAP IHC as a surrogate for CDKN2A deletion [36,38]. However, interpretation requires awareness of technical limitations and antibody variability. MTAP IHC provides information about protein expression, whereas molecular assays can characterize genomic alterations affecting MTAP and the neighboring CDKN2A locus. Neither measurement alone necessarily establishes the full functional consequences of MTAP deficiency or confirms sensitivity to a specific treatment. Moreover, differences in histological subtype, anatomical site, and intratumoral heterogeneity may influence the observed expression pattern and its interpretation.

Current evidence suggests that MTAP deficiency may be associated with more aggressive tumor behavior and shorter survival across human cancers. The evidence supporting the prognostic value of MTAP loss in PM is primarily derived from retrospective studies, most of which suggest that MTAP loss is an adverse prognostic factor associated with poorer survival, independently of other prognostic factors, including TP53 status. However, these findings are not entirely consistent, and the independent prognostic contribution of MTAP remains uncertain. Differences in cohort composition, histological subtype, anatomical site, assay methodology, and statistical adjustment may contribute to the variability across studies. Accordingly, the prognostic value of MTAP is biologically plausible and warrants further investigation. Additionally, the prognostic significance of MTAP loss in patients with peritoneal mesothelioma remains less well established.

Regarding the predictive value of MTAP loss, the available evidence is again derived primarily from retrospective studies. The predictive value of MTAP loss for response to chemotherapy remains controversial; however, overall, MTAP loss does not appear to be a reliable predictor of response to chemotherapy. Importantly, the potential association between MTAP loss and response to immunotherapy should be interpreted cautiously. Although retrospective studies have reported poorer outcomes among MTAP-deficient patients treated with immune-based therapies, these observations cannot establish MTAP loss as a predictive biomarker of immunotherapy resistance. In the absence of an appropriate treatment comparison group, it is not possible to determine whether the observed association reflects a treatment-specific effect or the underlying adverse prognostic impact of MTAP deficiency. Prospective biomarker-stratified studies, ideally including appropriate treatment comparison groups, are required to determine whether MTAP status has a treatment-specific predictive role in mesothelioma.

Clinical evidence supporting MTAP-directed therapies in mesothelioma remains preliminary. Early-phase studies of MTA-cooperative PRMT5 inhibitors have reported objective responses in patients with pleural mesothelioma, including two partial responses among the small mesothelioma subgroup treated with MRTX1719. However, these observations derive from very limited patient numbers without long-term follow-up and early-phase clinical studies and should therefore be interpreted as preliminary evidence of antitumor activity. Similarly, early clinical experience with AMG193 and MAT2A inhibitors has involved only small numbers of patients with mesothelioma, with limited evidence of objective tumor responses to date. Accordingly, although these findings provide clinical support for continued investigation of MTAP-directed strategies, their efficacy and clinical benefit in mesothelioma require confirmation in prospective studies with larger, molecularly characterized cohorts and longer follow-up.

Additional strategies discussed in this review, including targeting the Hippo–TEAD pathway and cell-cycle dependencies associated with CDKN2A loss, illustrate how the molecular context surrounding MTAP deficiency may reveal therapeutic opportunities beyond the direct inhibition of a single target. The relationship between MTAP status and the activity of TEAD or CDK4/6 inhibitors remains uncertain. Although a biological rationale exists for investigating these approaches in MTAP-deficient tumors, the available clinical data do not establish that their activity is specific to MTAP loss. Prospective studies with predefined biomarker-stratified analyses are required to determine whether MTAP status identifies patients who derive preferential benefit from TEAD or CDK4/6 inhibition.

Importantly, the clinical implications of MTAP loss should not be considered interchangeable between pleural and peritoneal mesothelioma. Most evidence supporting the diagnostic, prognostic, predictive, and therapeutic relevance of MTAP derives from pleural mesothelioma, whereas peritoneal mesothelioma shows a lower prevalence of MTAP loss, lower concordance between MTAP expression and CDKN2A status, and substantially more limited clinical evidence. Accordingly, MTAP status should be interpreted in an anatomical-site-specific context, and the prognostic, predictive, and therapeutic conclusions established in pleural mesothelioma require independent validation in peritoneal disease.

**Figure 2:**
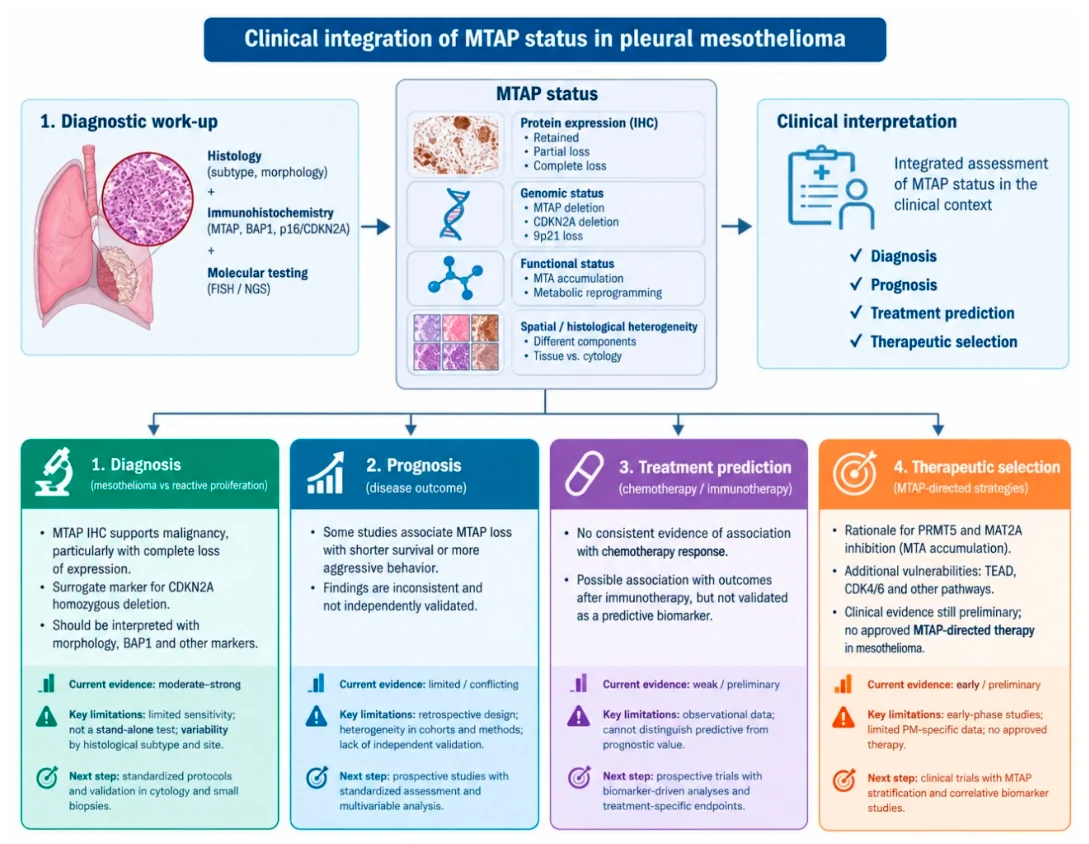
Clinical integration of MTAP status in PM.

Taken together, these findings suggest that the translational challenge is no longer simply to establish that MTAP loss is common in mesothelioma or that it provides a rationale for novel therapies. Rather, it is to define the conditions under which MTAP status provides clinically actionable information (Figure 2). For diagnostic purposes, the priority is to establish the accuracy and limitations of MTAP IHC in defined histological and cytological settings. For prognostic and predictive applications, the priority is to validate associations prospectively and determine whether MTAP adds information beyond established clinical and pathological variables. For therapeutic development, the priority is to establish whether molecular or functional evidence of MTAP deficiency identifies patients who derive a differential benefit from a specific treatment. Clinical trials of MTAP-associated therapeutic strategies should incorporate standardized biomarker assessment and report outcomes according to MTAP status, while accounting for relevant co-occurring alterations, including CDKN2A deletion.

## 10. Conclusions

In mesothelioma, MTAP loss represents a biologically relevant alteration associated with immune suppression, aggressive tumor behavior, and potential resistance to immune-based therapies. However, currently, MTAP loss is not an established predictive biomarker of immunotherapy resistance or TEAD/CDK4/6 inhibitor benefit. Future clinical trials should incorporate systematic assessment of MTAP status to better define the prognostic and predictive value of MTAP loss in PM. Preliminary results with MTA-cooperative PRMT5 inhibitors, MAT2A inhibitors, and other agents targeting the Hippo pathway suggest, for the first time, that targeted therapies may represent a potential therapeutic option for patients with PM. The results of ongoing clinical trials will be needed to determine the true efficacy of MTAP-directed therapies in a disease where effective treatments remain urgently needed.

## Figures and Tables

**Table 1:** Diagnostic approaches for MTAP loss in mesothelioma.

**Method**	**Advantages**	**Limitations**	**Clinical Relevance**
Immunohistochemistry	Rapid, widely available, inexpensive, applicable to biopsy and citology	Lack of standardized scoring criteria, variability among antibodies, interpretation affected by fixation and antigen retrieval	Increasingly used as a surrogate marker for CDKN2A deletion and tool to distinguish malignant proliferations from reactive lesions
FISH for CDKN2A/p16 deletion	Considered the reference method. Directly detects genomic deletion. High specificity	Requires specialized equipment. Expensive and time-consuming interpretation. Challenge in samples with low cellularity	Established molecular assay for distinguishing mesothelioma from benign mesothelial proliferations
FISH for MTAP deletion	Direct assessment of MTAP deletion. Useful for molecular confirmation	Requires specialized equipment. Less used than CDKN2A FISH	Useful for validation of MTAP IHC
Next-generation sequencing (NGS)	Simultaneous assessment of multiple genomic alterations	Variable sensitivity. Lack of universal thresholds for copy number alterations. High cost	Increasingly incorporated into molecular profiling of mesothelioma; may provide molecular confirmation of MTAP deletion when required for targeted therapy trial eligibility
Copy-number variation (CNV)	Wide assessment of deletion and co-alterations	Requires adequate quality and quantity of DNA	Useful in research
RNA expression analysis	Information on transcriptional consequences of genomic alterations	Expressions does not always correlate with genomic deletion	Useful in research

**Table 2:** Therapeutic strategies targeting MTAP.

**Therapy**	**Agent**	**Stage**	**PM (N)**	**Responses**
**Purine synthesis inhibition**	L-alanosine	Phase II	16	ORR 12%: 2 SD of 7 and 15 months
**PRMT5 transcriptional inhibition**	Quinacrine	Preclinical	N/A	N/A
**First-generation PRMT5 inh**	JNJ-64619175;	Preclinical/phase 1	2 (GSK)	ORR 0% Limiting toxiciy
	GSK3326595;			
	PF-06939999;			
	PRT543; PRT811			
**MTA-cooperative PRMT5 inh**	MRTX1719	Phase I	2	ORR 100%: 2 PR
**MTA-cooperative PRMT5 inh**	AMG193	Phase I	3	ORR 0%
**MTA-cooperative PRMT5 inh**	TNG908	Phase I/II	NR	NR
**MAT2A inh**	AG-270 (S095033)	Phase I	4	ORR 25%: 1 SD
**MAT2A inh**	IDE397	Phase I	NR	NR
**TEAD pathway inh**	ODM-212	Phase I	29	ORR 27%
**CDK4/6 inh**	Abemaciclib	Phase II	11 MTAP-loss PM	All tumor reduction

Abbreviations: Inh, inhibition; PM, pleural mesothelioma; ORR, objective response rate; PR, partial response; SD, stable disease; NR, not reported; N/A, not applicable.

**Table 3:** Current evidence, limitations, and clinical utility of MTAP in pleural mesothelioma.

**Clinical Question**	**Role of MTAP**	**Evidence**	**Current Limitation**
Mesothelioma vs. reactive proliferation	Diagnostic support	Relatively strong	Sensitivity is limited
CDKN2A deletion	Surrogate marker	Strong	Not fully interchangeable
Prognosis	Potential biomarker	Conflicting	Mostly retrospective
Chemotherapy response	Uncertain	Conflicting	No prospective validation
Immunotherapy response	Potential predictive biomarker	Preliminary	Requires prospective validation
PRMT5-directed therapy	Patient-selection biomarker	Increasing clinical evidence	No approved MTAP-directed therapy
MAT2A inhibition	Patient-selection biomarker	Early clinical evidence	Limited PM-specific data
TEAD/CDK4/6 strategies	Exploratory biomarker	Preliminary	Requires validation

## Data Availability

No new data were created or analyzed in this study. Data sharing is not applicable to this article.
